# Multiplatform Urinary Metabolomics Profiling to Discriminate Cachectic from Non-Cachectic Colorectal Cancer Patients: Pilot Results from the ColoCare Study

**DOI:** 10.3390/metabo9090178

**Published:** 2019-09-06

**Authors:** Jennifer Ose, Biljana Gigic, Tengda Lin, David B. Liesenfeld, Jürgen Böhm, Johanna Nattenmüller, Dominique Scherer, Lin Zielske, Petra Schrotz-King, Nina Habermann, Heather M. Ochs-Balcom, Anita R. Peoples, Sheetal Hardikar, Christopher I. Li, David Shibata, Jane Figueiredo, Adetunji T. Toriola, Erin M. Siegel, Stephanie Schmit, Martin Schneider, Alexis Ulrich, Hans-Ulrich Kauczor, Cornelia M. Ulrich

**Affiliations:** 1 Huntsman Cancer Institute, Salt Lake City, UT 84112, USA (J.O.) (T.L.) (J.B.) (H.M.O.-B.) (A.R.P.) (S.H.); 2 Department of Population Health Sciences, University of Utah, Salt Lake City, UT 84112, USA; 3 Department of General, Visceral and Transplantation Surgery, University of Heidelberg, 69117 Heidelberg, Germany (B.G.) (L.Z.) (P.S.-K.) (M.S.) (A.U.); 4 Division of Preventive Oncology, National Center for Tumor Diseases (NCT) and German Cancer Research Center (DKFZ), 69117 Heidelberg, Germany; 5 Diagnostic and Interventional Radiology, University of Heidelberg, 69117 Heidelberg, Germany (J.N.) (H.-U.K.); 6 Institute of Medical Biometry and Informatics, University of Heidelberg, 69117 Heidelberg, Germany; 7 European Molecular Biology Laboratory (EMBL), Genome Biology, 69117 Heidelberg, Germany; 8 Department of Epidemiology and Environmental Health, School of Public Health and Health Professions, University at Buffalo, Buffalo, NY 14260, USA; 9 Public Health Sciences Division, Fred Hutchinson Cancer Research Center, Seattle, WA 98109-1024, USA; 10 Department of Surgery, University of Tennessee Health Science Center, Memphis, TN 38163, USA; 11 Samuel Oschin Comprehensive Cancer Institute, Cedars-Sinai Medical Center, Los Angeles, CA 90048, USA; 12 Department of Surgery, Washington University School of Medicine and Siteman Cancer Center, St. Louis, MO 63110, USA; 13 Cancer Epidemiology Program, H. Lee Moffitt Cancer Center and Research Institute, Tampa, FL 33612, USA (E.M.S.) (S.S.)

**Keywords:** cancer cachexia, metabolomics, serial samples, urinary profiles, colorectal cancer

## Abstract

Cachexia is a multifactorial syndrome that is characterized by loss of skeletal muscle mass in cancer patients. The biological pathways involved remain poorly characterized. Here, we compare urinary metabolic profiles in newly diagnosed colorectal cancer patients (stage I–IV) from the ColoCare Study in Heidelberg, Germany. Patients were classified as cachectic (*n* = 16), pre-cachectic (*n* = 13), or non-cachectic (*n* = 23) based on standard criteria on weight loss over time at two time points. Urine samples were collected pre-surgery, and 6 and 12 months thereafter. Fat and muscle mass area were assessed utilizing computed tomography scans at the time of surgery. *N* = 152 compounds were detected using untargeted metabolomics with gas chromatography–mass spectrometry and *n* = 154 features with proton nuclear magnetic resonance spectroscopy. Thirty-four metabolites were overlapping across platforms. We calculated differences across groups and performed discriminant and overrepresentation enrichment analysis. We observed a trend for 32 compounds that were nominally significantly different across groups, although not statistically significant after adjustment for multiple testing. Nineteen compounds could be identified, including acetone, hydroquinone, and glycine. Comparing cachectic to non-cachectic patients, higher levels of metabolites such as acetone (Fold change (FC) = 3.17; *p* = 0.02) and arginine (FC = 0.33; *p* = 0.04) were observed. The two top pathways identified were glycerol phosphate shuttle metabolism and glycine and serine metabolism pathways. Larger subsequent studies are needed to replicate and validate these results.

## 1. Introduction

Cachexia is a multifactorial syndrome and affects 50–80% of all patients diagnosed with advanced cancer. The highest prevalence has been observed in cancers of the lung (83%) and the gastrointestinal tract (62%) [1]. Cachexia is defined as an ongoing loss of skeletal muscle mass that cannot be reversed by nutritional support and leads to progressive functional impairment [2,3,4]. Cancer patients typically progress from a pre-cachectic to a cachectic state over time (Table 1). Pre-cachectic cancer patients experience early clinical and metabolic symptoms (i.e., anorexia and impaired glucose tolerance) that may develop prior to involuntary weight loss [2]. Depending on cancer type and stage, pre-cachexia is further characterized by systemic inflammation, low food intake, and poor response to cancer treatment. Cachectic cancer patients experience similar, but more-intense, symptoms compared to pre-cachectic cancer patients including severe weakness, fatigue, and loss of muscle strength [2]. Prior studies have linked cancer cachexia to decreased quality of life, poor response to cancer treatment, and increased risk of death for various cancer types [5,6,7], including colorectal cancer [8,9,10,11]. Particularly obese cancer patients with low muscle mass, a state known as sarcopenic obesity, suffer from increased treatment-related toxicity and higher mortality rates in comparison to patients not diagnosed with sarcopenic obesity [12]. The loss of muscle mass in sarcopenic patients is frequently masked by a high body mass index (BMI). Thus, identifying these patients remains clinically challenging [12,13]. Biomarkers to identify cachexia independent of BMI may help to identify these patients early on. Furthermore, this may contribute to a better understanding of the mechanistic underpinnings of cachexia to develop efficient intervention methods that are lacking to date [2,3,4]. Additionally, the metabolic dysfunctions that lead to cancer cachexia are, as yet, poorly understood and thus, early detection of cachexia remains an unmet clinical need [14]. Biomarkers for early detection of muscle wasting and enhanced understanding of the molecular mechanisms leading to cachexia are urgently needed [15].

Here, we are expanding the results from two prior studies investigating the role of urinary metabolomics profiling, collected at three different time points, in prospectively followed colorectal cancer patients using an untargeted metabolomics approach [16,17]. We previously investigated the changes in urinary metabolic profiles of colorectal cancer patients at surgery, and at 6- and 12-month follow-ups, and identified microbial metabolites and described significant associations with disease stage [16]. In a subsequent analysis, we investigated the associations of urinary branched-chain amino acids (BCAAs) with parameters of energy balance and overall survival in colorectal cancer patients over time [17]. Branched-chain amino acids were not associated with body mass index (BMI), physical activity (metabolic equivalent [MET]), or muscle area (quantified by CT scans); however, BCAAs were independently associated with worse overall survival, particularly for patients diagnosed with stage IV colorectal cancer [17]. This study expands on our research interests in urinary metabolic profiles as a unique method to enhance our understanding of the etiology and progression of colorectal cancer.

The aim of this pilot project was to evaluate differences in urinary metabolomics profiles comparing non-cachectic, pre-cachectic, and cachectic colorectal cancer patients and to identify relevant metabolites and pathological pathways in the trajectory of cancer cachexia.

## 2. Results

We included a total of *n* = 27 newly diagnosed colorectal cancer patients. Table 1 shows the classification of colorectal cancer patients into cachexia and pre-cachexia based on the concept proposed by Fearon et al. [2]. Non-cachectic patients were defined as patients with ≤2% of weight loss in the past 6 months or weight gain, independent of BMI.

**Figure 1:**
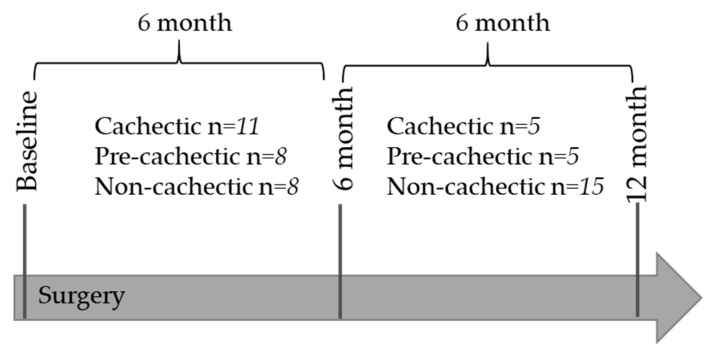
Classification of patients into the three groups: Between baseline and 6 months, and between 6 months and 12 months.

All patients were classified at two time points: Between baseline and 6 months, as well as between 6 months and 12 months (Figure 1). Cachexia status was calculated based on prospectively collected information on weight for each patient. Urine samples were collected at the same time points. Data from metabolomics analysis were available at all three time points. For statistical analyses, we used the metabolomics data generated from the second time point, i.e., if cachexia status was calculated from weight loss between 6 months and baseline, the 6 month measurement was used for statistical analyses.

Patient characteristics are presented in Table 2. Patients were mostly male, independent of cachexia phenotype (*p* = 0.33). Patients classified as cachectic or pre-cachectic were more often diagnosed with rectal cancer (both groups 62%), while 48% of non-cachectic patients were diagnosed with rectal cancer (*p* = 0.58). Distant metastasis (=stage IV) were only present in cachectic (25%) and pre-cachectic patients (15%), but not in non-cachectic patients (0%; *p* = 0.11). Cachectic patients (38%) were more likely to undergo neo-adjuvant therapy compared to non-cachectic patients (26%; *p* = 0.43). Adjuvant therapy was more likely to occur in cachectic and pre-cachectic patients (62%) compared to non-cachectic patients (30%; *p* = 0.14).

On an average, all patients were overweight at baseline with a mean BMI of 25.6 kg/m^2^ for cachectic patients; 29.4 kg/m^2^ for pre-cachectic patients, and 26.5 kg/m^2^ for non-cachectic patients with differences across the three groups (*p* = 0.02). As expected, we further observed differences in the amount of weight change (in kg) in a 6-month period across the three groups: Cachectic patients (–7.1 kg), pre-cachectic patients (−2.5 kg), and non-cachectic patients (+2.8 kg; *p* < 0.0001).

In Table 3, we compare body composition between cachectic, pre-cachectic, and non-cachectic patients. We observed nominally significant differences for visceral fat area (VFA; L4/L5) across the three groups (*p* = 0.04), comparing cachectic and pre-cachectic patients (*p* = 0.03) and pre-cachectic and non-cachectic patients (*p* = 0.04), respectively. No significant differences across the three groups or in two-group comparisons were observed for subcutaneous fat area on L3/L4 or L4/L5 (e.g., SFA; L4/L5; *p* = 0.19). Comparing dorsal muscle area, psoas muscle area, and abdominal muscle area on L3/L4 and L4/L5, we did not observe significant differences between the three groups or in two-group comparisons, except for a nominally significant difference of abdominal muscle area between cachectic and pre-cachectic patients on L3/L4 (*p* = 0.03).

We detected 152 features with gas chromatography–mass spectrometry (GC–MS) and 154 features with proton nuclear magnetic resonance (^1^H-NMR) spectrometry, respectively. We excluded 10 metabolites either from ^1^H-NMR or GC–MS datasets due to low Spearman rank correlation over the entire study population or serious overlap across spectra (malate, 3-hydroxyphenylacetate, vanillate, aspartate, creatinine, indole-3-acetate, 1-methylhystidine, phenylalanine, xanthine, and serine). A total of 34 metabolites were overlapping across the two platforms. We present Spearman correlation coefficients (crude) for all overlapping metabolites in Supplementary Materials Table S1.

Thirty-two compounds were nominally significantly different in group-wise comparisons. However, none of these results remained statistically significant after adjustment for multiple testing. Of these, 19 compounds were identified (Table 4, Table 5 and Table 6). Six metabolites differed in more than one group comparison, including three unknown metabolites: Acetone, arginine hydroquinone, glucuronide 29.801 min, sugar acid 15.570 min, and sugar acid 15.429 min. We compared metabolite concentrations by tumor stage across the phenotypes and observed overall comparable concentrations across tumor stages (Supplementary Table S2).

Comparing cachectic and non-cachectic patients (Table 4), eight metabolites had differing mean levels, with four being lower in cachectic patients (2.3.-butanediol, 2.3.- dihydroxybutyrate, acetone, and arginine) and four being higher (sugar 15.798 min, 4-hydroxyphenylacetate, glucuronide 28.543 min, and 3-methylxanthine) compared to patients without cachexia. Comparing cachectic and pre-cachectic patients (Table 5), 12 metabolites were different between the two groups. Of these, nine were lower (hydroquinone, aminomalate, sugar acid 15.429 min, 4-hydroxy-3-methoxy-mandelate, glucuronide 29.801 min, sugar acid 15.750 min, sugar 14.575 min, disaccharide 29.943 min, and glycine) and three were higher (one unknown at 13.271 min, isobutyrate and ethleneglycol) in cachetic compared to pre-cachectic patients. Comparing pre-cachectic and non-cachectic patients (Table 6), 19 metabolites had differing mean levels in the two groups. Of these, 15 were higher (such as tartrate, hydroquinone, and several glucuronides) and three were lower in pre-cachectic compared to non-cachectic patients (uracil, acetone, and arginine). None of these differences were significant after adjustment for multiple testing.

**Figure 2:**
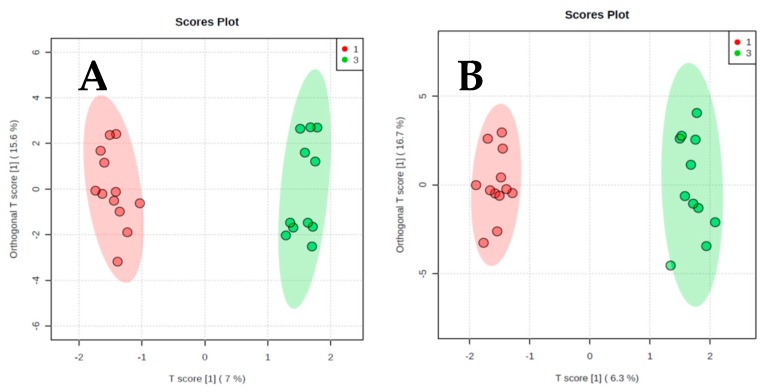
Orthogonal projections to latent structures discriminant analysis. (**A**) Cachectic patients and non-cachectic patients. Urinary Metabolites only. (**B**) Cachectic and non-cachectic patients. Urinary metabolites and information on fat and muscle area from CT scans.

**Figure 3:**
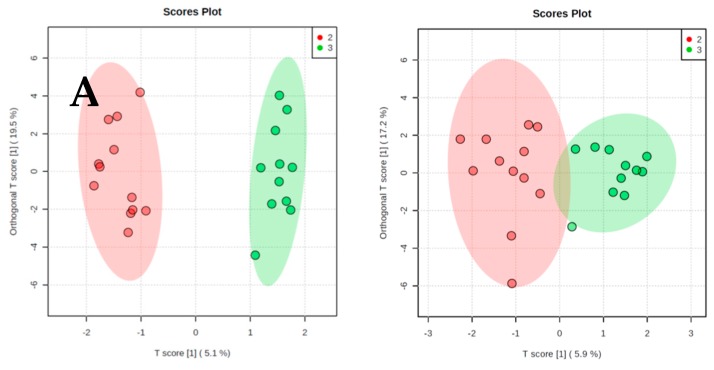
Orthogonal projections to latent structures discriminant analysis. (**A**) Pre-cachectic patients and non-cachectic patients. Urinary Metabolites only. (**B**) Pre-cachectic patients and non-cachectic patients. Urinary metabolites and information on fat and muscle area from CT scans.

**Figure 4:**
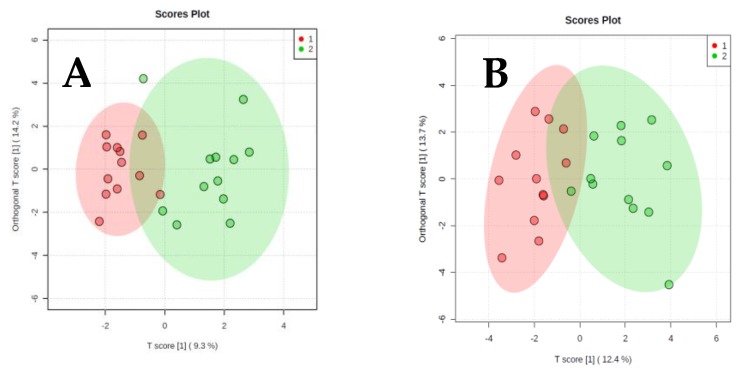
Orthogonal projections to latent structures discriminant analysis. (**A**) Pre-cachectic patients and cachectic patients. Urinary Metabolites only. (**B**) Pre-cachectic patients and cachectic patients. Urinary metabolites and information on fat and muscle area from CT scans.

The results of orthogonal partial least-squares discriminant analysis (OPLS-DA) are presented in Figure 2, Figure 3 and Figure 4. We present two score plots for each comparison, one including solely the identified metabolites and one with additional information on visceral and subcutaneous fat area as well as muscle area. Using urinary metabolites only, we were able to successfully discriminate cachectic from non-cachectic patients (Figure 2A), and pre-cachectic from non-cachectic patients (Figure 3A), but not pre-cachectic from cachectic patients (Figure 4A). Adding the information on fat and muscle mass area did not improve discrimination in these group comparisons. For example, the score for the comparison of cachectic and non-cachectic patients was only slightly different when data on fat and muscle mass were included: Orthogonal T score (15.6% *versus* 16.7%) and a slightly lower T score (7.0% *versus* 6.3%; Figure 2B).

**Figure 5:**
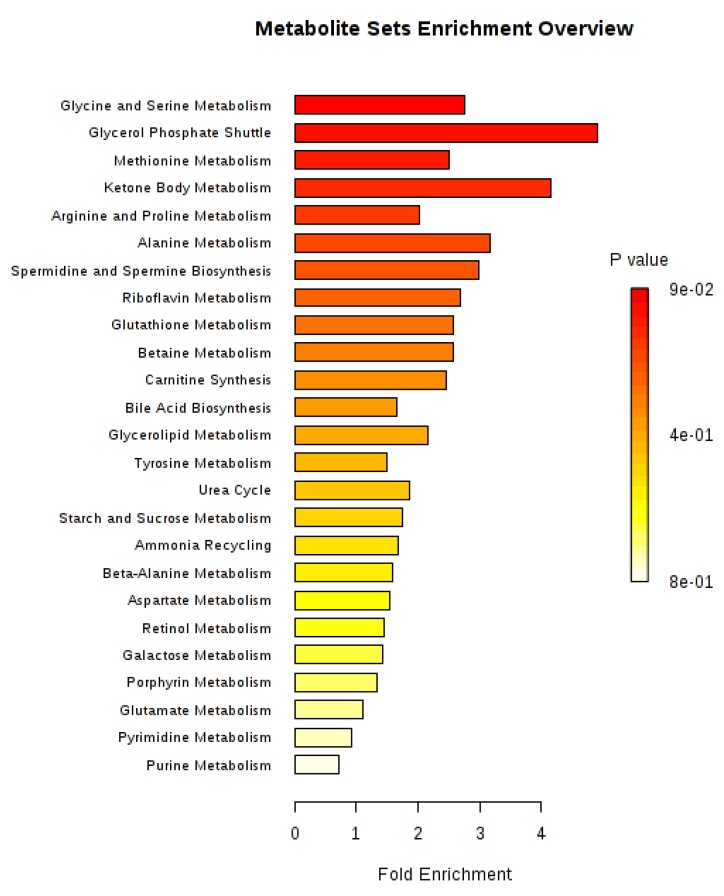
Summary plot for over-representation analyses (ORA). Overall urinary metabolites. For each of the listed pathways the respective metabolites identified are listed below: Glycine and serine metabolism: Glycine, L-arginine, L-methionine (3/53); glycerol phosphate shuttle: Hydroquinone (1/11); methionine metabolism: Glycine, L-methionine (2/43); ketone body metabolism: Acetone (1/13); arginine and proline metabolism: L-arginine (1/35); alanine metabolism: Glycine, L-arginine (2/53); spermidine and spermine biosynthesis: L-methionine (1/18); riboflavin metabolism: Hydroquinone (1/20); glutathione metabolism: Glycine (1/21); betaine metabolism: L-methionine (1/21); carnitine synthesis: Glycine (1/22); bile acid biosynthesis: Glycine, cholic acid (2/65); glycerolipid metabolism: Hydroquinone (1/25); tyrosine metabolism: p-Hydroxyphenylacetic acid, aminomalate (2/72); urea cycle: L-arginine (1/29); starch and sucrose metabolism: Sucrose (1/31); ammonia recycling: Glycine (1/32); beta-alanine metabolism: Uracil (1/34); aspartate metabolism: L-arginine (1/35); retinol metabolism: Glucoronide (1/37); galactose metabolism: Sucrose (1/38); porphyrin metabolism: Glycine (1/40); glutamate metabolism: Glycine (1/50); pyrimidine metabolism: Uracil (1/59); purine metabolism: Glycine (1/74).

**Figure 6:**
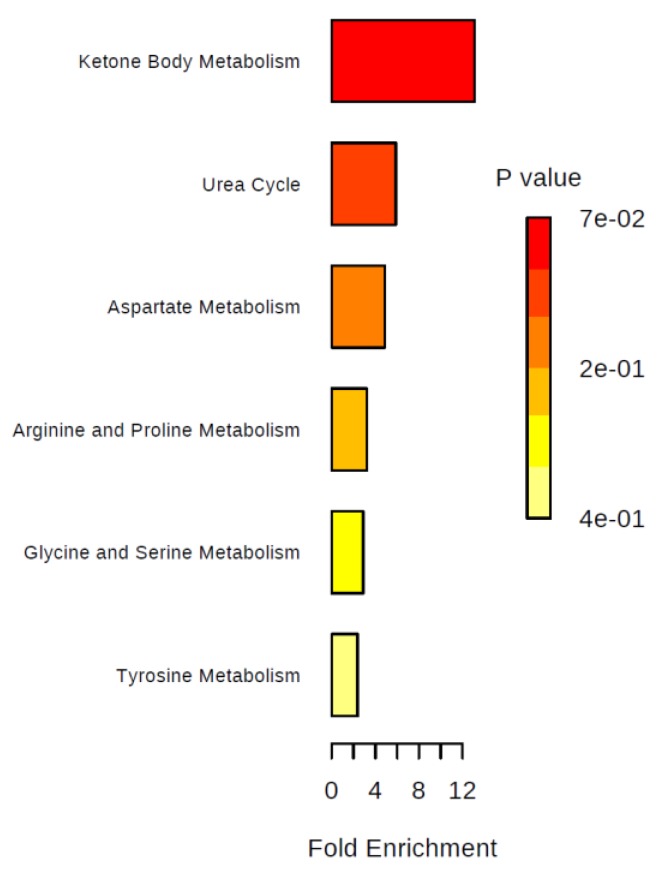
Summary plot for over-representation analyses (ORA): Urinary metabolites that were different between cachectic and non-cachectic patients. For each of the listed pathways, the respective metabolites identified are listed below: Ketone body metabolism: Acetone (1/13); urea cycle: Arginine (1/29); aspartate metabolism: L-arginine (1/35); arginine and proline metabolism: L-arginine (1/35); glycine and serine metabolism: L-arginine (1/59); tyrosine metabolism: p-Hydroxyphenylacetic acid (1/72).

**Figure 7:**
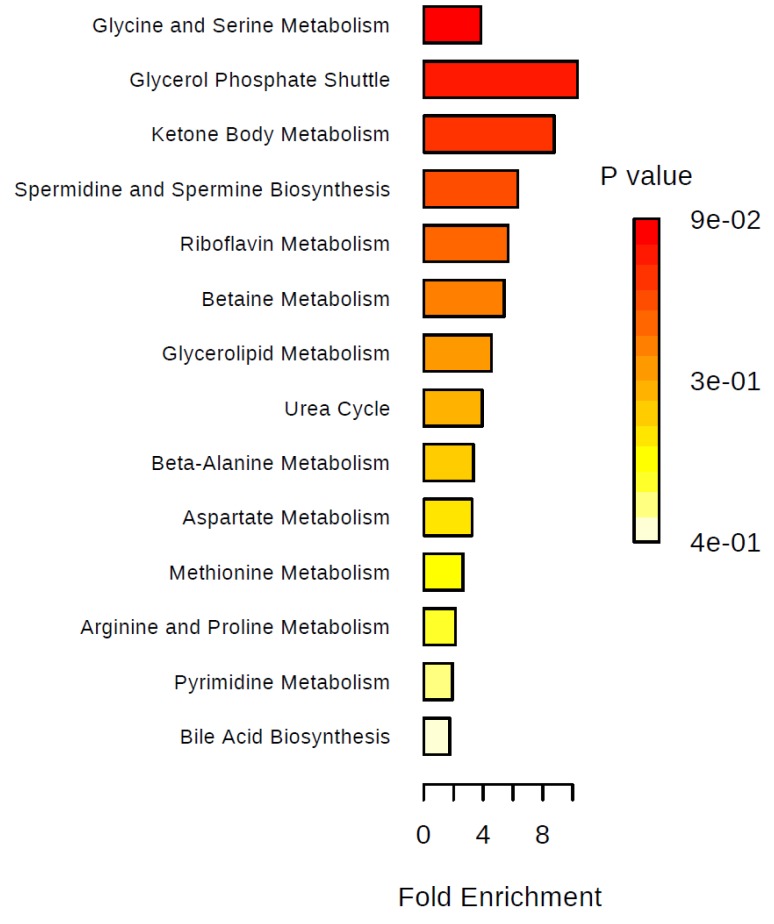
Summary plot for over-representation analyses (ORA). Metabolites that were different between pre-cachectic and non-cachectic patients. For each of the listed pathways, the respective metabolites identified are listed below: Glycine and serine metabolism: L-arginine and L-methionine (2/59); glycerol phosphate shuttle: Hydroquinone (1/11); ketone body metabolism: Acetone (1/13); spermidine and spermine biosynthesis: L-methionine (1/18); riboflavin metabolism: Hydroquinone (1/20); betaine metabolism: L-methionine (1/21); glycerolipid metabolism: Hydroquinone (1/25); urea cycle: L-arginine (1/29); beta-alanine metabolism: Uracil (1/34); aspartate metabolism: L-arginine (1/35); methionine metabolism: L-methionine (1/43); arginine and proline metabolism: L-arginine (1/53); pyrimidine metabolism: Uracil (1/59); bile acid biosynthesis: Cholic acid (1/65).

**Figure 8:**
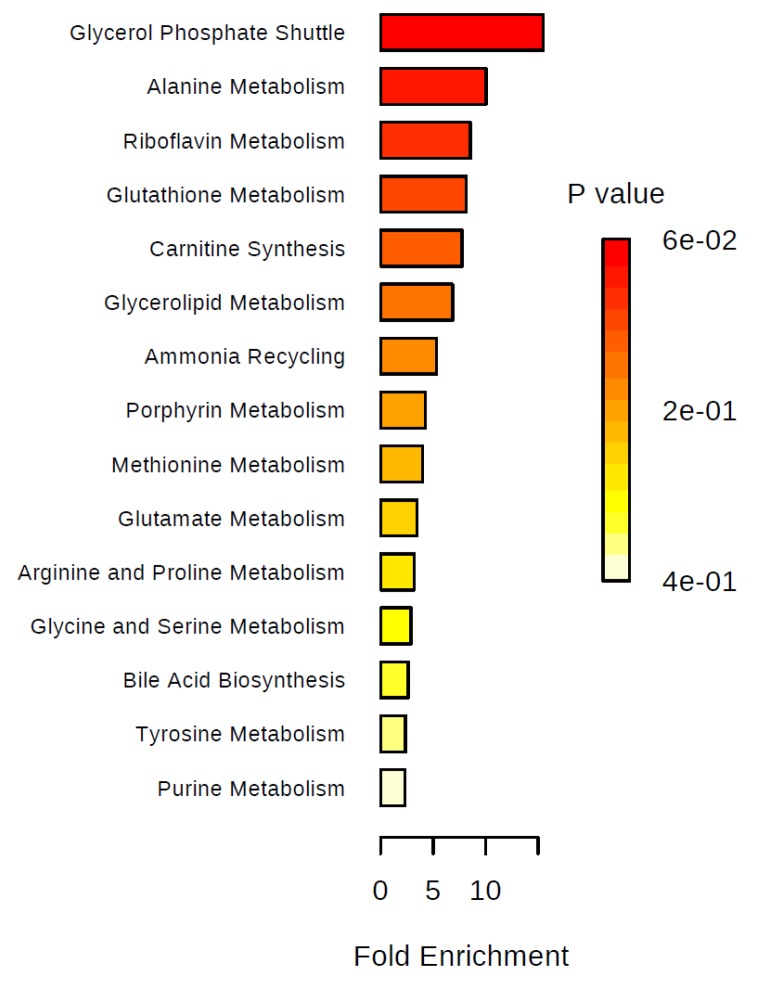
Summary plot for over-representation analyses (ORA). Metabolites that were different between pre-cachectic and cachectic patients. For each of the listed pathways, the respective metabolites identified are listed below: Glycerol phosphate shuttle: Hydroquinone (1/11); alanine metabolism: Glycine (1/17); riboflavin metabolism: Hydroquinone (1/20); glutathione metabolism: Glycine (1/21); carnitine synthesis: Glycine (1/22); glycerolipid metabolism: Hydroquinone (1/25); ammonia recycling: Glycine (1/32); porphyrin metabolism: Glycine (1/40); methionine metabolism: Glycine (1/43); glutamate metabolism: Glycine (1/49); arginine and proline metabolism: Glycine (1/53); glycine and serine metabolism: Glycine (1/59); bile acid biosynthesis: Glycine (1/65); tyrosine metabolism: Aminomalonate (1/72); purine metabolism: Glycine (1/74).

We performed over-representation analyses overall and in two-group comparisons (Figure 5, Figure 6, Figure 7 and Figure 8). Using all identified metabolites, the top three pathways identified were: Glycerol phosphate shuttle, ketone body metabolism, and glycine and serine metabolism (Figure 5). Using the metabolites that were nominally significantly different between pre-cachectic and non-cachectic patients, the same three pathways were identified (Figure 6). For the comparison between cachectic and pre-cachectic patients, glycerol phosphate shuttle, and alanine and riboflavin metabolism were identified (Figure 8). The top pathway comparing cachectic and non-cachectic patients was ketone body metabolism (Figure 7).

## 3. Discussion

This is the largest study to date aiming to discriminate cachectic from non-cachectic colorectal cancer patients (*n* = 27) using an untargeted metabolomics approach. Thirty-two metabolites were nominally significantly different across the three phenotypes; of those, 19 were classified (level 1 and 2) and linked to glycerol phosphate shuttle metabolism and glycine and serine metabolism pathways. Adding information on fat and muscle area did not improve discrimination.

Three prior studies used metabolomics to identify specific biomarkers and pathways that are related to cancer cachexia utilized urine or serum, or both biospecimens with GC–MS, LC–MS, or ^1^H-NMR [18,19,20]. Two studies combined several cancer types under the assumption that cachexia-related pathways are independent of cancer types, while one study focused on pancreatic cancer.

Fujiwara et al. examined intra-day variation of serum metabolites in a group of advanced pancreatic cancer cases, 9 of which were classified as cachectic and 12 as non-cachectic using Eastern Cooperative Oncology Group (ECOG) guidelines [18]. In a pilot study, serum samples from *n* = 8 cachectic and *n* = 7 non-cachectic patients diagnosed with various cancer types (including one non-cachectic colon cancer patient) were analyzed on three different metabolomic platforms: GC–MS (70 metabolites), LC–MS (244 metabolites), and CE–MS (232 metabolites) [19]. Yang et al. measured metabolites in serum and urine samples from *n* = 306 cancer patients and healthy controls, using a discovery-validation approach on the ^1^H-NMR platform.

This study included a total of *n* = *21* colorectal cancer patients [18]. In summary, these prior studies identified similar metabolites as the current study, including acetone, arginine, and glycine, as well as pathways such as glycine and serine metabolism.

In the present study, we observed increased concentrations of glycine in cachectic *versus* non-cachectic patients. This is in line with the studies from Cala et al. [19] and Yang et al. [18]. High serum concentrations of glycine have been previously associated with metastatic colorectal cancer among patients who had cachexia [21]. Glycine supplementation in vivo has been shown to protect against muscle loss in obese mice on caloric restriction [22]. There is further evidence that mitochondrial dysfunction in skeletal muscle leads to increased cellular glycine production, which in turn is protective against muscle wasting conditions, such as cachexia (reviewed in [22,23]). One of the major pathways identified in the present study was the glycine and serine pathway, which has also been described by the two prior studies that included various cancer types [18,19]. Unfortunately, we had to exclude serine from the analyses due to severe spectral overlap and we were unable to replicate the findings from prior studies [18,19].

We observed higher arginine concentrations in cachectic compared to non-cachectic patients, similar to the study by Yang et al. [18] Arginine is a semi-essential amino acid and can be synthesized from glutamine [24]. Dual functions have been described for arginine in the context of cancer: As an onco-nutrient essential for cancer growth, as well as an immuno-nutrient enhancing immune response (as reviewed in [25]). Arginine metabolism has been linked to several cancer types that rely on extracellular arginine to maintain rapid tumor growth [24,26]. In vitro studies have shown that colorectal cancer cells quickly deplete intracellular arginine stores [26]. In a murine model, arginine supplementation decreased colorectal tumor incidence and overall colorectal tumor burden when supplemented early in the disease course [25].

In our study, we also observed that hydroquinone was higher in cachectic compared to non-cachectic patients, but lower in pre-cachectic compared to non-cachectic patients. Hydroquinone (1,4-benzenediol) is a benzene metabolite. None of the prior studies have related hydroquinone to cancer cachexia. However, the study by Fujiwara et al. in pancreatic cancer patients has described significant intra-day variations of p-hydroxybenzoic acid comparing cachectic and non-cachectic patients. Hydroquinone is a result of enzymatic biodegradation of p-hydroxybenzoic acid. Prior in vitro and in vivo studies have reported anti-cancer activity of hydroquinone [27]. The pathway related to hydroquinone—glycerol 3 phosphate shuttle pathway—is further related to glycolysis, fatty acid metabolism, and oxidative phosphorylation [28]. Mitochondrial glycerol-3-phosphate dehydrogenase (mGPDH), which was identified in the present study, is a basic element of the mitochondrial respiratory chain. mGDPH is the rate-limiting step in the glycerol 3 phosphate shuttle pathway [29]. It further attenuates skeletal muscle regeneration in vitro and in vivo by controlling mitochondrial biogenesis [30].

We observed a >3-fold increase in acetone comparing cachectic and non-cachectic patients, similar to the results published by Yang and colleagues [18]. Acetone belongs to the group of endogenous ketone bodies and is produced by the spontaneous breakdown of acetoacetate. All cells with mitochondria can use these ketone bodies and reconvert them into acetyl-CoA. Acetyl-CoA is used as subsequent fuel in citric acid cycles. Ketone bodies are usually absent in the urine of healthy individuals, but their presence is indicative of fat being used as an energy source instead of carbohydrates. High concentrations of acetone in the blood during starvation can lead to ketosis, and in its extreme form to ketoacidosis. Pleiotropic effects of acetone including the regulation of inflammation, oxidative stress, and anti-catabolic effects in human skeletal muscle have been described previously [31]. The observed associations between metabolites and different cachexia phenotypes may reflect subclinical changes in muscle metabolism and the use of fat as source of energy instead of carbohydrates.

Larger subsequent studies will allow consolidation of the results. A particular strength of this study is the use of a non-invasive approach for continuous disease monitoring [32]. Plasma measurements have been shown to be poor indicators of skeletal muscle metabolism [9]. However, in a recent study in patients diagnosed with advanced cancer, urine metabolites predicted skeletal muscle mass with high accuracy (Pearson *r* = 0.98; *p* < 0.0001) and therefore, were selected as the best matrix for the present study [10].

Differences between the present and prior studies may be due to differential classification of cachexia and slightly different categorization of BMI. We considered individuals who had a weight change of ±5% and a BMI >20 kg/m^2^ within 6 months according to the predominantly used method by Fearon et al. [2], while Yang et al. classified non-cachectic patients as individuals that are weight-stable: ‘No marked weight change’ in the prior year and a BMI <25 kg/m^2^. BMI values, as well as cancer stage, were not presented, so it is difficult to see how our sample differs from Yang et al. [16]. Another source for differences between this and prior studies may be the use of different metabolomics platforms. The present study focused on GC–MS and ^1^H-NMR, while other studies used LC–MS. Given the underlying technical differences between these analytical platforms, the chemical diversity of metabolites varies; thus, each platform provides a specific metabolomics profile.

This study has strengths and limitations. We used state-of-the-art metabolomics platforms, namely GC–MS and ^1^H-NMR, for the measurement of urinary metabolites. A specific advantage of ^1^H-NMR is that sample preparation does not require extra steps, which enables very accurate measurement of compounds. Missing chromatographic separation of metabolites and limited sensitivity are known to complicate compound identification. GC–MS offers combined sensitivity and selectivity platforms for metabolomics profiling studies. However, identification of detectable peaks using GC–MS is, in general, less complete compared to ^1^H-NMR. The present study did not include LC–MS analysis. It is possible some relevant metabolites may have been omitted because of this approach. Although we observed 32 metabolites that were significantly different (nominal *p*-values) between the different phenotypes, the majority of compounds are classified as unknowns (level 3 and 4). The identification of these compounds continues to be a challenge in metabolomics studies in general [33]. None of the identified metabolites that were different across groups were robust after adjustment for multiple testing in this pilot study. While this may be the largest to date, the group sizes were small and there were a lot of multiple comparisons done. The significance did not remain after adjustment for multiple comparisons. At the Heidelberg ColoCare study site, we measured the muscle tissue using a threshold limit of 40 to 100 HU to measure selectively the dense, functional relevant part without fatty infiltration. In the literature, however, a wider threshold (−29 to 150 HU) is used to measure the total muscle tissue. Thus, the available CT scan data could not be reliably utilized for assessing sarcopenia [12,34,35]. In the present study we observed that patients defined as pre-cachectic had higher BMI and higher visceral adipose tissue area compared to the two other phenotypes. It appears that criteria that are solely based on weight loss over time may not be adequate to define pre-cachexia. Additionally, using information on sarcopenia may have resulted in a more accurate classification of patients.

Glycine is a metabolite that is known to be detected via GC–MS, as well as ^1^H-NMR. In the present study, glycine was detected via ^1^H-NMR but not GC–MS. Although we are not entirely sure, we speculate that this discrepancy could potentially be related to the derivatization proc.

Furthermore, metabolite concentration may vary according to internal as well as external confounders that could not be adequately addressed due to small sample size.

The results of this pilot study, however, justify larger studies to investigate the mechanistic underpinnings of cancer cachexia. Currently larger efforts are ongoing in the ColoCare Study to conduct longitudinal analysis of metabolomics biomarker data and trajectories across cachexia groups.

## 4. Materials and Methods

The ColoCare Study is a multicenter international prospective cohort recruiting newly diagnosed colorectal cancer patients (stages I through IV; International Classification of Diseases, 10th edition, C18–C20) and aiming to investigate predictors of cancer recurrence, treatment toxicities, survival, and health-related quality of life (ClinicalTrials.gov: NCT02328677) [16,36,37].

The present pilot study was performed in *n* = 52 patients recruited at the ColoCare site in Heidelberg, Germany between October 2010 and January 2013. After study approval by the Institutional Review Board of the Medical Faculty at the University of Heidelberg (IRB identification code 310/2001), written informed consent was obtained from all participants.

Demographic information, as well as data on established risk factors, were collected using standardized questionnaires at surgery, and at 6 and 12 months thereafter. BMI was measured and calculated as kg/m^2^ at each time point. Baseline examination includes questionnaires, anthropometric measurements, assessment of physical activity, and biospecimen collection (e.g., blood and urine samples). Information on adjuvant chemotherapy and medical history were abstracted from clinical records. Patients’ survival data was monitored both by access to medical records and as part of the active patient follow-up within ColoCare. If patients died, the time point of death was documented.

We defined cases as cachectic (*n* = 18) or pre-cachectic (*n* = 16) for colorectal cancer patients based on the predominantly used criteria by Fearon et al. [2] (Table 1). We further selected *n* = 11 non-cachectic cancer patients (e.g., cancer patients with stable weight over 6 months).

### 4.1. Patient Selection and Classification

In the present study, we included patients that had data available on urinary biospecimens, GC–MS, and ^1^H-NMR measures, as well as weight measurements at all three time points: Before surgery (baseline), and at 6 months and 12 months after surgery. Patients were defined as cachectic or pre-cachectic based on weight change in the past 6 months, either between (i) baseline and 6 month or (ii) 6 month and 12 month time points (Table 1, Figure 1) [2]. Non-cachectic cancer patients were selected from the same study population and included if they had <2% of weight loss in the past 6 months, independent of their BMI. For option (i), urinary measurements at the 6-month follow-up were used for statistical analyses, and for option (ii), urinary measurements at the 12-month follow-up were used for statistical analyses.

### 4.2. Biospecimen Collection

Urinary samples were collected at the National Center of Tumor Diseases (NCT), Heidelberg at three time points—prior to surgery (*n* = 97) and in some cases 1–8 days after surgery (*n* = 12), and 6 months (*n* = 52) and 12 months (*n* = 38) after surgery. If patients underwent adjuvant chemotherapy, follow-up visits were scheduled at least 2 weeks after their last chemotherapy cycle had been completed. Urine samples were aliquoted immediately and stored at −80 °C until analysis. Self-reported food and fluid intake before sample collection was monitored during follow-up visits. Eligible patients were then selected based on the availability of urinary metabolomics data and at least two subsequent time points where weight had been measured.

### 4.3. Laboratory Analyses and Quality Control Measurements

We used gas chromatography–mass spectrometry (GC–MS) and proton nuclear magnetic resonance spectroscopy (^1^H-NMR) for the metabolomics profiling of urinary samples, as described in detail previously [16].

### 4.4. Sample Preparation and Quality Control: GC–MS

Preparation of urine samples for GC–MS was based on the method by Cheng and colleagues [11]. Urine samples were thawed and vortexed briefly and aliquots of 50 μL were transferred into a reaction tube and 10 μL internal standard solution was added. The sample was transferred into an amber vial with micro inserts and subjected to GC–MS analysis. We used pooled quality control (QC) samples and Kovats retention mixtures (50 μg*mL^−1^) injected with every analytical batch. A LOESS function was used to correct drifts for each metabolite and relative standard deviations (%RSD) were used to estimate the analytical noise. In addition, we analyzed paired samples (samples from one patient collected at three time points: Pre-surgery, and 6 and 12 months after surgery) in the same analytical batches to account for time-dependent bias due to analytical drifts. Analyses were carried out on an Agilent 6890 GC/ 5973 MS single quadrupole system. Data acquisition was started after a solvent delay of 5 min. Spectra were acquired over a range of 50–500 m/z [16].

### 4.5. Sample Preparation and Quality Control: ^1^H-NMR

Sample preparation for ^1^H-NMR was performed according to the method by Xiao and colleagues: 540 μL urine was spiked with 60 μL K_2_HPO_4_/NaH_2_PO_4_ buffer in D_2_O (pH 6.5, 1.5 M). [38] QC samples were run at the beginning and end of each analytical batch. Citrate was measured in duplets during all data acquisitions and a 14.7% RSD in the raw ^1^H-NMR data was indicative of an analytically robust method and a short-term variance (across *n* = 100 samples) of a median %RSD of 5%.

### 4.6. Data Pre-Processing

GC–MS: Raw files were converted to netCFD format, imported into MZMine 2.0 [39], and chromatograms crop filtered (5–32 min) and baseline corrected. Masses were detected with centroid algorithm and chromatogram building was carried out with minimum time span of 0.1 min, minimum height of 1E3, and mass accuracy 0.5 m/z. For peak chromatogram deconvolution, noise amplitude of 1E3, a time span of 0.05–1.5 min, and a peak minimum of 1E4 were chosen. Peaks were aligned with the join aligner with 0.5 m/z mass accuracy and 0.1 min time tolerance, and a 50:50 weight for mass and retention time. Gap filling was performed with 1% tolerance at 0.5 m/z mass accuracy and 0.1 min [retention time] tolerance. Artifact peaks resulting from the derivatization process and interferences due to urea were removed from the data set. Since glutamate and pyroglutamate are interconvertible during GC–MS derivatization, these metabolites were combined. The retention time (Rt), mass spectrometric base peak used for quantification, confidence level in identification, matches those listed in the Human Metabolome Database (HMDB) and PubChem.

^1^H NMR: Spectra were processed automatically using the Bruker TopSpin software with zero-filling (6-fold), the application of 0.3 Hz of line broadening, phase correction, and referenced relative to TSP incorporated as an internal standard [16]. Processed data files were subsequently imported in batch mode into the dataChord spectrum miner software (OneMoon Scientific, Westfield, NJ, USA). Each region was integrated and the total integral area of each bin divided by the area of the internal standard TSP creating the final data table for statistical analysis. The spectroscopic bins for which resonances of urinary metabolites were reported, based on the Chenomx Library version 7.7 / HMDB.

Metabolite annotation: We annotated chromatographic peaks based on four different levels: Level 1: Authentic reference standard available; level 2: Electron impact (EI) spectral match (>75%) and Kovats retention indices (RI) match (20) to the NIST 2011 database; level 3: Only EI spectral match (>75%) to multiple components of a chemical class (i.e., sugar acids); level 4: Unknown metabolite.

### 4.7. Normalization of Metabolite Concentrations

Concentrations of metabolites in urine are dependent on hydration status and fluid intake. In order to account for these factors, metabolite concentrations in the urine were normalized. The raw peak area of GC–MS data was normalized by sum, log-transformed, and auto-scaled prior to analysis. ^1^H-NMR data were normalized to creatinine (µmol/mmol creatinine) and auto-scaled [40].

### 4.8. Area-Based Computed Tomography (CT) Quantification of Abdominal Adipose Tissue and Muscle Tissue Area

We have described the quantification of abdominal adipose tissue area [41] and muscle tissue area previously [17]. In brief, abdominal CT scans conducted between August 2010 and December 2014 were assessed retrospectively using Centricity RIS 4.1i and GE PACS (GE Medical Systems, Buckinghamshire, UK). CT scans were predominantly performed before surgery (mean time, before: 42 days, after: 41 days). A prior study that used data from the same population showed that pre- and post-surgical CT scans were similar, and thus, could be combined for analyses. [42] The quantification of VFA and SFA based on diagnostic CT data was performed using a dedicated post-processing software (Syngo Volume tool, MMPW, Siemens Healthcare, Munich, Berlin, Germany). An area-based quantification of adipose tissue compartments was performed on two spinal levels most representative of the abdominal adipose tissue distribution (L3/L4, L4/L5) using a lower threshold of –190 HU and an upper threshold of –30 HU to selectively measure adipose tissue [42]. The area of the *dorsal* muscles, abdominal muscles, and muscle *psoas major* were also quantified using abdominal CT scans [42] CTs from pre- and post-surgery were similar and thus, combined for statistical analyses [42]. Specific regions of interest (ROI) of muscle area (*dorsal muscles, abdominal muscles,* and the muscle *psoas major*) were manually determined and measured on the same levels (vertebral body L3/4 and L4/5) by a volumetric tool (MMWP, Syngo Volume tool, Siemens Healthcare, Munich, Berlin, Germany) [42,43]. The measurement limits for aforementioned selected ROIs were at a minimum attenuation of 40 Hounsfield Units (HU) to a maximum of 100 HU to selectively measure muscle tissue by avoiding muscles’ lipid content [35].

### 4.9. Statistical Analysis

Data processing of urine metabolite measures from ^1^H-NMR and GC–MS were conducted separately using the commercially available Metaboanalyst software 4.0 [44].

Mean and standard deviations were calculated for continuous variables. Data on weight, height, and BMI were available at all three time points. Fold change was calculated for all two-group comparisons using GC–MS data, logFoldChange was calculated for all two-group comparisons using ^1^H-NMR data. We used the Benjamini–Hochberg procedure to control the false discovery rate (FDR) and to account for multiple testing [45].

One-way ANOVA was applied for multiple group comparison. Pearson’s Chi-squared test was conducted for testing differences in categorical variables. Pearson’s partial correlation coefficients were calculated, adjusting for sex and age at surgery. We used over-representation enrichment analysis and excluded metabolites from the analysis that have not been detected by the two detection systems (GC–MS, ^1^H-NMR) to avoid biases, as some metabolites sets may be overrepresented by the nature of the detection systems. Orthogonal partial least-squares discriminant analysis (OPLS-DA) was used to investigate if urinary metabolomics can discriminate cachectic from pre-cachectic or non-cachectic patients.

Statistical analyses and figure-plotting were performed using SAS for Windows, version 9.4 and the Metaboanalyst software [44]. All tests were two-sided with significance level 0.05.

## 5. Conclusions

The metabolic profiling of urinary samples across multiple metabolomics platforms revealed differences in glycerol phosphate shuttle and glycine and serine pathways in cachectic, pre-cachectic, and non-cachectic colorectal cancer patients. These results need to be replicated in larger cohorts. Using a non-invasive untargeted metabolomics approach may enable the identification of biomarkers for early detection of cachexia and could potentially be used for continuous monitoring of cancer patients in the clinic.

## Figures and Tables

**Table 1:** Criteria for classification of patients as cachectic or pre-cachectic.

Category	Weight Loss	BMI
Non-Cachectic	weight loss ≤ 2% weight gain	-
Pre-Cachectic	weight loss * ≤ 5%	BMI ≤ 20 kg/m^2^
Cachectic	weight loss * > 5%	
Cachectic	weight loss * > 2%	BMI < 20 kg/m^2^

* in the past 6 months. BMI, body mass index.

**Table 2:** Description of cachectic, pre-cachectic, and non-cachectic patients.

	Cachectic (*n* = 16)	Pre-Cachectic (*n* = 13)	Non-Cachectic (*n* = 23)	*p*-Value
**Age at surgery ^+^**	58.38 (±10.33)	55.84 (±11.67)	62.74 (±12.22)	0.21 ^+^
**BMI ^1^** (kg/m^2^)				
at Baseline ^2,+^	25.60 (±2.71)	29.39 (±3.35)	26.48 (±4.26)	0.02 ^+^
6 Months afterwards ^3,+^	23.37 (±3.08)	28.11 (±3.42)	26.54 (±4.41)	0.004 ^+^
**Weight Change (kg) ^+^**	−7.12 (±3.63)	−2.54 (±1.27)	2.78 (±2.68)	<0.001 ^+^
**Weight Change (%)**	−0.09 (±0.05)	−0.02 (±0.02)	0.03 (±0.03)	<0.001 ^+^
**Sex**				0.33 ^++^
Male	11 (69%)	11 (85%)	14 (61%)	
Female	5 (31%)	2 (15%)	9 (39%)	
**Stage**				0.11 ^++^
I	5 (31%)	2 (15%)	7 (30%)	
II	1 (6%)	5 (38%)	9 (40%)	
III	6 (38%)	4 (31%)	7 (30%)	
IV	4 (25%)	2 (15%)	0	
**Site**				0.58 ^++^
Colon	6 (38%)	5 (38%)	12 (52%)	
Rectal	10 (62%)	8 (62%)	11(48%)	
**Adjuvant Therapy**				0.14 ^++^
Yes	10 (62%)	8 (62%)	7 (30%)	
No	6 (38%)	5 (38%)	16 (70%)	
**Neo-Adjuvant Therapy**				0.43 ^++^
Yes	6 (38%)	4 (31%)	6 (26%)	
No	10 (62%)	9 (70%)	17 (74%)	

^1^ BMI = body mass index; ^2^ Baseline as first assessment, either pre-surgery or at 6-month follow-up; ^3^ follow-up at 6 months after pre-surgery assessment or at 6-month follow-up. ^+^ One-way ANOVA was used to compare mean differences across the three different phenotypes for continuous variables. ^++^ Pearson’s Chi-square test was used to test for distribution differences in categorical variables across the three different phenotypes.

**Table 3:** Body composition at baseline in cachectic, pre-cachectic, and non-cachectic patients. ^+^

	Cachectic (*n* = 16)	Pre-Cachectic	Non-Cachectic	*p*-Value for Group Comparisons			
		(*n* = 13)	(*n* = 23)	Three Group Comparison	Cachectic vs Non-Cachectic	Cachectic vs Pre-Cachectic	Pre-Cachectic vs Non-Cachectic
**Visceral Fat Area**							
L3/L4	152.93 (±92.56)	231.20 (±68.97)	184.26 (±68.59)	0.08	0.34	0.04	0.11
L4/L5	134.90 (±74.86)	204.51 (±57.86)	154.96 (±51.81)	0.04	0.44	0.03	0.04
**Subcutaneous Fat Area**							
L3/L4	204.58 (±101.90)	301.70 (±125.96)	255.47 (±130.21)	0.20	0.30	0.06	0.39
L4/L5	230.00 (±97.10)	320.38 (±111.91)	286.10 (±125.43)	0.19	0.23	0.06	0.50
***Dorsal muscle* Area**							
L3/L4	37.77 (±14.86)	30.31 (16.34)	35.43 (17.25)	0.57	0.72	0.29	0.47
L4/L5	29.44 (±10.26)	21.84 (14.66)	27.93 (11.34)	0.32	0.73	0.18	0.26
***Psoas muscle* Area**							
L3/L4	19.30 (±8.57)	18.55 (±6.19)	19.34 (±6.86)	0.96	0.98	0.82	0.77
L4/L5	28.81 (±24.44)	19.29 (±6.58)	22.30 (±7.93)	0.34	0.36	0.25	0.34
***Abdominal muscle* Area**							
L3/L4	42.88 (±17.46)	26.02 (±14.07)	34.25 (±15.12)	0.06	0.20	0.03	0.19
L4/L5	36.07 (±14.49)	24.92 (±14.58)	30.01 (±15.61)	0.24	0.33	0.09	0.43

^+^ One-way ANOVA was used to compare mean differences across the three different phenotypes for continuous variables.

**Table 4:** Comparison of mean level of metabolites in cachectic and non-cachectic patients. Intensities of the raw peak area of a certain ion (GC–MS) were normalized by sum, log-transformed, and auto-scaled and each spectral bin (^1^H-NMR) was normalized to creatinine (µmol/mmol creatinine) and auto-scaled. ^+^

Platform	Metabolite (mean ± std)	Cachectic	Non-Cachectic	Fold Change	*p*_Value_^1^	*p*_FDR_^2^	Identification
**GC–MS ^3^**	2.3-Butanediol	0.2 ± 0.76	1.0 ± 0.73	0.45	0.01	0.93	Level 2
	2.3-Dihydroxybutyrate	0.1 ± 0.59	−0.5 ± 0.56	1.82	0.02	0.93	Level 2
	Sugar 15.798 min	0.3 ± 1.02	1.1 ± 0.89	0.45	0.04	0.93	Level 3
	4-Hydroxyphenylacetate	−0.2 ± 0.67	0.5 ± 0.98	0.50	0.04	0.93	Level 2
	Unknown Glucuronide 28.543 min	0.3 ± 0.66	0.9 ± 0.98	0.55	0.04	0.93	Level 3
**^1^H-NMR ^4,5^**	3-Methylxanthine	3.3 ± 1.81	5.8 ± 2.87	0.76	0.02	0.97	NMR library
	Acetone	7.1 ± 7.37	1.7 ± 1.01	3.17	0.02	0.97	NMR library
	Arginine	25.8 ± 6.66	19.4 ± 8.03	0.33	0.04	0.97	NMR library

^+^ One-way ANOVA was used to compare mean differences across different phenotypes for continuous variables. ^1^ *p*_Value_: *p*-value for the comparison of mean levels across cachectic and non-cachectic patients; ^2^ *p*_FDR_: *p*-value from Benjamini–Hochberg procedure; ^3^ GC–MS: Gas chromatography–mass spectrometry; ^4 1^H-NMR: Proton nuclear magnetic resonance; ^5^ for ^1^H-NMR values were not log transformed, thus we present log fold change values.

**Table 5:** Comparison of mean level of metabolites in cachectic and pre-cachectic patients. Intensities of the raw peak area of a certain ion (GC–MS) were normalized by sum, log-transformed, and auto-scaled and each spectral bin (^1^H-NMR) was normalized to creatinine (µmol/mmol creatinine) and auto-scaled.^+^

Platform	Metabolite (Mean ± Std)	Cachectic	Pre-Cachectic	Fold Change	*p*_Value_^1^	*p*_FDR_^2^	Identification
**GC-MS ^3^**	Hydroquinone	0.6 ± 0.49	−0.5 ± 1.20	3.00	0.006	0.59	Level 1
	Unknown 13.271 min	0.0 ± 0.86	0.8 ± 0.80	0.45	0.009	0.59	Level 4
	Aminomalonate	0.4 ± 0.59	−0.3 ± 0.77	2.01	0.01	0.59	Level 2
	Sugar Acid 15.429 min	0.5 ± 0.74	−0.1 ± 0.55	1.82	0.01	0.60	Level 3
	4-Hydroxy-3-Methoxy-Mandelate	0.6 ± 1.15	−0.3 ± 0.83	2.46	0.03	0.79	Level 2
	Unknown Glucuronide 29.801 min	0.7 ± 0.79	0.0 ± 1.06	2.01	0.03	0.79	Level 3
	Sugar Acid 15.750 min	0.6 ± 0.79	−0.1 ± 1.16	2.01	0.04	0.79	Level 3
	Unknown Sugar 14.575 min	0.4 ± 0.94	−0.4 ± 1.23	2.23	0.04	0.79	Level 3
	Unknown Dissacharide 29.943 min	0.5 ± 0.76	−0.2 ± 1.12	2.01	0.04	0.79	Level 3
**^1^H-NMR ^4,5^**	Isobutyrate	0.8 ± 0.78	1.7 ± 1.21	1.13	0.02	0.97	NMR library
				0.79			
	Glycine	150.1 ± 118.51	72.5 ± 42.98	1.07E+33	0.04	0.97	NMR library

^+^ One-way ANOVA was used to compare mean differences across different phenotypes for continuous variables. ^1^ *p*_Value_: *p*-value for the comparison of mean levels across cachectic and non-cachectic patients; ^2^ *p*_FDR_: *p*-value from Benjamini–Hochberg procedure; ^3^ GC–MS: Gas chromatography–mass spectrometry; ^4 1^H-NMR: Proton nuclear magnetic resonance; ^5^for ^1^H-NMR values were not log transformed, thus we present log fold change values.

**Table 6:** Comparison of mean level of metabolites in pre-cachectic and non-cachectic patients. Intensities of the raw peak area of a certain ion (GC–MS) were normalized by sum, log-transformed, and auto-scaled and each spectral bin (^1^H-NMR) was normalized to creatinine (µmol/mmol creatinine) and auto-scaled. ^+^

Platform	Metabolite (Mean ± Std)	Pre-Cachectic	Non-Cachectic	Fold Change	*p*_Value_^1^	*p*_FDR_^2^	Identification
**GC-MS ^3^**	Sugar Acid 15.429 min	−0.1 ± 0.55	0.8 ± 0.86	0.41	0.007	0.59	Level 3
	2-O-Glycerol-α-d-galactopyranoside	−0.4 ± 1.09	0.6 ± 0.49	0.37	0.01	0.59	Level 2
	Sugar 15.798 min	0.0 ± 1.06	1.1 ± 0.89	0.33	0.01	0.59	Level 3
	Tartrate	0.0 ± 0.85	1.0 ± 1.07	0.37	0.01	0.59	Level 1
	Hydroquinone	−0.5 ± 1.20	0.7 ± 1.14	0.30	0.02	0.59	Level 1
	Sugar Acid 15.750 min	−0.1 ± 1.16	0.8 ± 0.61	0.41	0.03	0.59	Level 3
	Unknown 28.636 min	0.1 ± 1.14	1.1 ± 0.94	0.37	0.03	0.59	Level 4
	Unknown Glucuronide 27.116 min	0.0 ± 0.83	0.7 ± 0.67	0.50	0.03	0.59	Level 3
	Unknown Glucuronide 28.543 min	0.1 ± 0.89	0.9 ± 0.98	0.45	0.04	0.59	Level 3
	Sugar 14.052 min	−0.4 ± 1.34	0.7 ± 1.02	0.33	0.04	0.59	Level 3
	p-cresol-glucuronide	−0.3 ± 1.27	0.6 ± 0.68	0.41	0.04	0.59	Level 1
	Unknown Glucuronide 27.688 min	0.1 ± 0.86	0.9 ± 0.75	0.45	0.04	0.59	Level 3
	Unknown Glucuronide 29.801 min	0.0 ± 1.06	0.8 ± 0.90	0.45	0.049	0.59	Level 3
**^1^H-NMR ^4,5^**	Uracil	6.5 ± 2.10	3.9 ± 2.15	0.67	0.008	0.91	NMR library
	Cholate	0.8 ± 0.12	1.6 ± 0.27	1.00	0.03	0.91	NMR library
	Methionine	4.4 ± 2.54	2.2 ± 1.37	1.00	0.03	0.91	NMR library
	Acetone	3.2 ± 1.83	1.7 ± 1.01	0.88	0.03	0.91	NMR library
	3-Phenylpropionate	9.1 ± 3.97	14.4 ± 6.05	0.58	0.03	0.91	NMR library
	Arginine	28.7 ± 11.98	19.4 ± 8.03	0.48	0.045	0.91	NMR library

^+^ One-way ANOVA was used to compare mean differences across different phenotypes for continuous variables. ^1^ *p*_Value_: *p*-value for the comparison of mean concentrations across cachectic and non-cachectic patients; ^2^ *p*_FDR_: *p*-value from Benjamini–Hochberg procedure; ^3^ GC–MS: Gas chromatography–mass spectrometry, ^4 1^H-NMR: Proton nuclear magnetic resonance; ^5^ for ^1^H-NMR values were not log transformed, thus we present log fold change values.

## Supplementary Materials

The following are available online at https://www.mdpi.com/2218-1989/9/9/178/s1, Table S1: Spearman correlations for metabolites overlapping across GC–MS and ^1^H-NMR*; Table S2: Comparison of metabolite levels across cachectic, pre-cachectic, and non-cachectic patients by tumor stage. One-way ANOVA was used to assess the *p* for difference across the three groups.
