# Supplementary material for: Multiplatform Urinary Metabolomics Profiling to Discriminate Cachectic from Non-Cachectic Colorectal Cancer Patients: Pilot Results from the ColoCare Study
Source: Metabolites. 2019 Sep 6;9(9):178. doi: 10.3390/metabo9090178 (PMC6780796; doi:10.3390/metabo9090178)
Supplement: Supplementary file 1 [file metabolites-09-00178-s001.zip › metabolites-562632-supp-final/SupplementaryTable S2.pdf]

Supplementary Table S2. Comparison of metabolite levels across cachectic, pre-cachectic and non-cachectic patients by tumor stage. One-way anova was used to assess the p for difference across the three groups.

	Tumor Stage	Cachectic	Pre-cachectic	Non-cachectic	pvalue
**2-O-Glycerol-alpha-d-galactopyranoside**	Stage I	0.1 ± 1.29	-0.3 ± 0.92	0.6 ± 0.50	0.97
	Stage II	-0.1 ±0.00	-0.6 ± 0.79	0.6 ± 0.60	0.49
	Stage III	0.2 ± 0.82	-0.3 ± 1.86	0.5 ± 0.19	0.30
	Stage IV	-0.3 ± 1.07	-0.1 ± 0.07	NA	0.28
**p-cresol-glucuronide**	Stage I	0.2 ± 1.28	0.8 ± 0.22	0.3 ± 0.64	0.30
	Stage II	0.1 ± 0.00	-0.5 ± 0.89	0.8 ± 0.77	0.31
	Stage III	0.1 ± 0.88	0.1 ± 0.30	0.5 ± 0.63	0.77
	Stage IV	0.2 ± 0.71	-2.0 ± 2.48	NA	0.63
**Unknown Glucuronide 27.116min**	Stage I	1.6 ± 1.76	0.4 ± 0.21	0.3 ± 0.90	0.67
	Stage II	0.3 ± 0.00	-0.4 ± 0.97	0.7 ± 0.50	0.13
	Stage III	0.2 ± 0.58	0.5 ± 0.80	1.4 ± 0.41	0.45
	Stage IV	0.5 ± 0.79	-0.4 ± 0.02	NA	0.54
**Unknown Glucuronide 27.688min**	Stage I	0.6 ± 1.15	0.5 ± 0.01	0.6 ± 0.62	0.09
	Stage II	0.1 ± 0.00	-0.3 ± 1.21	1.0 ± 0.81	0.69
	Stage III	0.2 ± 0.78	0.4 ± 0.48	0.6 ± 1.01	0.75
	Stage IV	1.2 ± 1.25	0.5 ± 0.87	NA	0.39
**Unknown Glucuronide 28.543min**	Stage I	0.6 ± 0.61	0.1 ± 0.06	0.6 ± 0.67	0.97
	Stage II	-0.8 ± 0.00	-0.6 ± 1.04	1.0 ± 1.22	0.64
	Stage III	0.0 ± 0.60	0.4 ± 0.25	1.4 ± 0.83	0.21
	Stage IV	0.6 ± 0.47	1.2 ± 0.56	NA	0.84
**Unknown 28.636min**	Stage I	0.9 ± 1.14	-0.1 ± 0.71	1.6 ± 0.46	0.07
	Stage II	0.3 ± 0.00	-0.6 ± 0.69	0.8 ± 1.16	0.32
	Stage III	0.3 ± 0.30	0.2 ± 1.04	1.0 ± 0.63	0.22
	Stage IV	0.9 ± 0.64	1.7 ± 1.55	NA	0.42
**2.3-Dihydroxybutyrate**	Stage I	0.1 ± 0.60	-0.5 ± 1.07	-0.4 ± 0.55	0.03
	Stage II	0.8 ± 0.00	0.4 ± 1.40	-0.6 ± 0.64	0.92
	Stage III	0.2 ± 0.75	-0.3 ± 1.09	-0.1 ± 0.18	0.63
	Stage IV	-0.1 ± 0.31	-0.3 ± 0.37	NA	0.05

**Unknown Glucuronide 29.801min**	Stage I	0.9 ± 1.02	0.3 ± 0.93	0.8 ± 1.20	0.36
	Stage II	0.4 ± 0.00	-0.4 ± 1.26	0.7 ± 0.99	0.30
	Stage III	0.4 ± 0.49	-0.1 ± 1.11	1.1 ± 0.01	0.12
	Stage IV	1.1 ± 0.96	0.6 ± 0.83	NA	0.06
**Unknown Dissacharide 29.943min**	Stage I	1.1 ± 0.56	-0.2 ± 0.85	-0.6 ± 1.37	0.26
	Stage II	0.9 ± 0.00	-0.4 ± 0.95	0.2 ± 0.99	0.02
	Stage III	0.0 ± 0.72	-0.4 ± 1.73	1.3 ± 1.35	0.57
	Stage IV	0.4 ± 0.63	0.6 ± 0.19	NA	0.22
**Arginine**	Stage I	28.4 ± 6.54	24.8 ± 13.17	15.0 ± 13.84	0.74
	Stage II	37.9 ± 0.00	27.9 ± 6.99	21.8 ± 4.24	0.40
	Stage III	22.9 ± 5.52	27.5 ± 19.53	19.7 ± 6.21	0.70
	Stage IV	23.9 ± 5.64	37.3 ± 4.70	NA	0.13
**Isobutyrate**	Stage I	1.1 ± 1.54	3.6 ± 0.49	1.4 ± 1.88	0.13
	Stage II	0.8 ± 0.00	1.4 ± 1.12	0.7 ± 0.47	0.004
	Stage III	0.7 ± 0.22	1.5 ± 0.78	2.1 ± 0.58	0.02
	Stage IV	0.7 ± 0.29	0.9 ± 1.21	NA	0.12
**Methionine**	Stage I	6.8 ± 6.52	3.0 ± 2.54	1.9 ± 1.24	0.05
	Stage II	2.8 ± 0.00	3.4 ± 0.73	2.8 ± 1.47	0.08
	Stage III	2.5 ± 0.73	4.8 ± 2.66	1.7 ± 1.82	0.11
	Stage IV	3.2 ± 1.18	7.4 ± 4.43	NA	0.15
**2.3-Butanediol**	Stage I	0.5 ± 1.02	0.5 ± 1.07	0.6 ± 0.80	0.11
	Stage II	0.1 ± 0.00	-0.4 ± 0.96	0.9 ± 0.50	0.11
	Stage III	0.2 ± 0.74	0.1 ± 1.49	1.9 ± 0.68	0.32
	Stage IV	0.0 ± 0.62	0.9 ± 2.93	NA	0.37
**3-Phenylpropionate**	Stage I	11.9 ± 3.91	6.3 ± 0.18	16.3 ± 1.96	0.07
	Stage II	12.0 ± 0.00	9.8 ± 3.82	13.4 ± 8.78	0.45
	Stage III	9.7 ± 6.90	10.1 ± 5.53	12.7 ± 0.00	0.66
	Stage IV	10.2 ± 6.13	8.4 ± 0.00	NA	0.76
**Cholate**	Stage I	1.4 ± 1.32	NA	1.5 ± 0.24	
	Stage II	NA	0.7 ± 0.00	1.8 ± 0.00	0.70
	Stage III	2.1 ± 2.54	NA	NA	0.51

	Stage IV	1.6 ± 0.00	0.9 ± 0.00	NA	0.13
**Ethyleneglycol**	Stage I	7.3 ± 4.39	12.1 ± 4.09	9.6 ± 7.56	0.18
	Stage II	9.0 ± 0.00	12.3 ± 7.12	16.1 ± 9.37	0.01
	Stage III	11.1 ± 4.54	24.8 ± 11.81	13.7 ± 8.16	0.14
	Stage IV	8.5 ± 3.47	11.8 ± 0.44	NA	0.65
**Hydroquinone**	Stage I	0.8 ± 0.64	0.5 ± 0.16	-0.4 ± 1.69	0.07
	Stage II	-0.2 ± 0.00	-1.1 ± 1.23	0.9 ± 0.54	0.09
	Stage III	0.8 ± 0.26	-0.4 ± 1.43	1.5 ± 0.34	0.118
	Stage IV	0.4 ± 0.37	-0.3 ± 0.95	NA	0.014
**3-Methylxanthine**	Stage I	1.4 ± 0.58	5.0 ± 2.93	5.3 ± 1.17	0.08
	Stage II	1.3 ± 0.00	5.8 ± 4.09	6.6 ± 3.90	0.38
	Stage III	4.1 ± 1.66	1.8 ± 0.99	4.4 ± 1.71	0.09
	Stage IV	4.1 ± 1.53	2.9 ± 0.16	NA	0.03
**Acetone**	Stage I	8.7 ± 9.54	2.7 ± 1.41	1.1 ± 0.69	0.48
	Stage II	6.9 ± 0.00	3.4 ± 2.65	2.0 ± 0.99	0.49
	Stage III	5.9 ± 4.06	2.6 ± 1.12	2.2 ± 1.49	0.59
	Stage IV	6.9 ± 10.80	4.7 ± 0.59	NA	0.72
**Aminomalonate**	Stage I	0.4 ± 0.52	0.1 ± 0.48	-0.7 ± 1.39	0.83
	Stage II	0.5 ± 0.00	-0.1 ± 0.18	0.0 ± 0.64	0.14
	Stage III	0.4 ± 0.77	-0.8 ± 0.98	0.6 ± 0.37	0.83
	Stage IV	0.3 ± 0.60	-0.1 ± 1.60	NA	0.04
**Glycine**	Stage I	84.7 ± 20.05	48.7 ± 27.73	51.9 ± 40.18	0.41
	Stage II	268.0 ± 0.00	67.3 ± 23.50	124.7 ± 126.98	0.19
	Stage III	183.8 ± 165.65	63.3 ± 25.83	172.1 ± 148.66	0.3
	Stage IV	144.1 ± 107.67	127.7 ± 97.47	NA	0.76
**Uracil**	Stage I	5.6 ± 2.09	8.6 ± 0.92	3.5 ± 2.80	0.03
	Stage II	9.2 ± 0.00	6.1 ± 1.48	3.7 ± 1.35	0.13
	Stage III	6.2 ± 3.27	4.8 ± 1.42	4.9 ± 3.96	0.91
	Stage IV	2.5 ± 0.99	8.9 ± 2.16	NA	
**Unknown 13.271min**	Stage I	-0.3 ± 0.61	0.4 ± 1.48	0.8 ± 1.15	0.10
	Stage II	0.7 ± 0.00	0.8 ± 0.96	-0.1 ± 0.72	0.14

	Stage III	-0.1 ± 0.96	1.1 ± 0.52	0.7 ± 0.55	0.22
	Stage IV	0.2 ± 1.15	0.8 ± 0.63	NA	0.39
**4-Hydroxyphenylacetate**	Stage I	0.3 ± 0.64	0.5 ± 0.61	-0.3 ± 0.37	0.73
	Stage II	0.3 ± 0.00	0.1 ± 1.52	0.8 ± 1.12	0.52
	Stage III	-0.4 ± 0.59	0.8 ± 0.64	1.1 ± 0.46	0.56
	Stage IV	-0.6 ± 0.64	0.0 ± 1.67	NA	0.20
**Tartrate**	Stage I	1.2 ± 1.70	0.3 ± 0.07	1.2 ± 1.13	0.61
	Stage II	0.2 ± 0.00	-0.2 ± 1.10	0.7 ± 0.83	0.53
	Stage III	0.0 ± 0.51	0.1 ± 0.99	1.9 ± 1.77	0.53
	Stage IV	-0.2 ± 0.28	-0.1 ± 0.76	NA	0.91
**Sugar 14.052min**	Stage I	0.3 ± 1.87	-0.7 ± 0.33	0.6 ± 0.36	0.09
	Stage II	-0.2 ± 0.00	0.0 ± 0.59	0.5 ± 1.34	0.001
	Stage III	-0.2 ± 0.64	-0.2 ± 2.28	1.4 ± 0.15	0.68
	Stage IV	0.6 ± 0.87	-1.4 ± 1.25	NA	0.53
**Unknown Sugar 14.575min**	Stage I	0.2 ± 0.35	-0.2 ± 0.32	-0.7 ± 2.35	0.70
	Stage II	-2.2 ± 0.00	0.3 ± 1.22	-0.1 ± 0.78	0.74
	Stage III	0.9 ± 0.83	-0.9 ± 1.55	-0.2 ± 0.07	0.84
	Stage IV	0.8 ± 0.21	-1.2 ± 0.34	NA	0.13
**Sugar Acid 15.429min**	Stage I	1.2 ± 0.86	-0.1 ± 0.18	0.7 ± 0.18	0.22
	Stage II	-0.6 ± 0.00	-0.4 ± 0.66	0.4 ± 0.67	0.51
	Stage III	0.5 ± 0.38	0.3 ± 0.23	1.8 ± 1.44	0.27
	Stage IV	0.2 ± 0.42	0.0 ± 0.88	NA	0.39
**Sugar Acid 15.750min**	Stage I	0.9 ± 0.68	-0.1 ± 1.64	0.7 ± 0.94	0.58
	Stage II	-1.0 ± 0.00	-0.8 ± 0.57	0.8 ± 0.62	0.77
	Stage III	0.8 ± 0.68	0.1 ± 1.24	0.7 ± 0.05	0.05
	Stage IV	0.4 ± 0.74	1.0 ± 1.52	NA	0.68
**Sugar 15.798min**	Stage I	0.8 ± 1.32	0.4 ± 0.91	1.7 ± 1.62	0.11
	Stage II	-1.1 ± 0.00	-0.6 ± 1.04	0.9 ± 0.45	0.83
	Stage III	0.3 ± 0.62	0.4 ± 0.75	0.9 ± 0.51	
	Stage IV	0.0 ± 1.07	0.5 ± 1.83	NA	0.28
**4-Hydroxy-3-Methoxy-Mandelate**	Stage I	0.8 ± 0.91	-0.1 ± 0.21	-0.6 ± 0.50	0.33

	Stage II	-0.6 ± 0.00	-0.6 ± 0.50	0.3 ± 0.75	0.79
	Stage III	0.6 ± 1.67	-0.4 ± 1.34	-0.1 ± 1.19	0.86
	Stage IV	0.5 ± 0.60	0.3 ± 0.80	NA	0.006
