# Supplementary material for: Multiplatform Urinary Metabolomics Profiling to Discriminate Cachectic from Non-Cachectic Colorectal Cancer Patients: Pilot Results from the ColoCare Study
Source: Metabolites. 2019 Sep 6;9(9):178. doi: 10.3390/metabo9090178 (PMC6780796; doi:10.3390/metabo9090178)
Supplement: Supplementary file 1 [file metabolites-09-00178-s001.zip › metabolites-562632-supp-final/SupplementaryTableS1.pdf]

Supplementary Table S1. Spearman correlations for metabolites overlapping across GC-MS and ^{1}H-NMR.*

	N	Correlation Coefficient	pValue
Hippurate	37	0.51	**0.001**
Alanine	37	0.48	**0.002**
Hypoxanthine	37	0.46	**0.004**
Valine	37	0.43	**0.008**
4-Hydroxyphenylacetate	34	0.41	**0.02**
Citrate	37	0.39	**0.02**
Homovanillate	36	0.36	**0.03**
myo-Inositol	36	0.36	**0.03**
3-Hydroxyisobutyrate	37	0.35	**0.03**
IndoIe-3-acetate	36	0.34	**0.04**
Threonine	37	0.30	0.07
Xanthurenate	26	-0.34	0.08
4-Hydroxy-3-methoxymandelate	29	-0.32	0.08
Arabinose	37	0.28	0.09
2-Hydroxyphenylacetate	20	0.37	0.10
Mannitol	37	0.23	0.17
Glutamine	37	0.20	0.23
Tyrosine	37	0.16	0.34
Histidine	37	0.12	0.46
N-Acetylglucosamine	44	-0.10	0.47
Glutamate	36	0.11	0.53
Glucose	37	-0.10	0.53
Glycolate	37	-0.09	0.56
Tartrate	37	0.07	0.65
3-Aminoisobutyrate	36	0.14	0.69
Pyroglutamate	37	-0.13	0.73
Asparagine	37	-0.06	0.74
5-Hydroxyindole-3-acetate	31	0.03	0.87
Gluconate	37	-0.03	0.87
Lactate	37	-0.01	0.95
Fructose	36	-0.01	0.97
2-Hydroxyglutarate	3	NA	
2-Phosphoglycerate	3	NA	
Lysine	1	NA	

*significant pValues are bold
